# Pemetrexed Plus Lenalidomide for Relapsed/Refractory Primary Central Nervous System Lymphoma: A Prospective Single-Arm Phase II Study

**DOI:** 10.3389/fonc.2022.938421

**Published:** 2022-07-11

**Authors:** Jingjing Ma, Zhiguang Lin, Tianling Ding, Qing Li, Mengxue Zhang, Hui Kang, Patrick B. Johnston, Yan Ma, Bobin Chen

**Affiliations:** ^1^ Department of Hematology, Huashan Hospital, Fudan University, Shanghai, China; ^2^ Department of Hematology, Huashan Hospital North, Fudan University, Shanghai, China; ^3^ Division of Hematology, Department of Internal Medicine, Mayo Clinic, Rochester, MN, United States

**Keywords:** primary central nervous system lymphoma, relapsed/refractory, pemetrexed, lenalidomide, efficacy and safety

## Abstract

The prognosis of relapsed/refractory (R/R) primary central nervous system lymphoma (PCNSL) is dismal, and there are limited treatment options for these patients. This was a prospective single-arm phase II study of combined pemetrexed and lenalidomide for salvage treatment of R/R PCNSL. Patients with R/R PCNSL (n = 38) who had undergone two or more different therapeutic regimens and experienced disease progression or recurrence were enrolled. The primary endpoint was overall response rate (ORR). Secondary endpoints were progression-free survival (PFS) and overall survival (OS). Patients were followed up for a median of 18 (range, 1–36) months. ORR was 68.4%, with median PFS and OS of 6 and 18 months, respectively. Adverse events (AEs) included myelosuppression, fatigue, nausea, fever, infection, cardiac disease, and thrombogenesis. Commonly observed grade ≥ 3 AEs included neutropenia (5.3%), leukopenia (2.6%), thrombocytopenia (7.9%), and infection (2.6%). Elevated lactate dehydrogenase (LDH) levels (χ^2^ = 13.25; P = 0.0003) and bulky disease (P = 0.032; χ^2^ = 4.580) were associated with short PFS. Elevated serum LDH level (P = 0.011; χ^2^ = 6.560), abnormal lymphoma cells in the cerebrospinal fluid (CSF) [P = 0.011; χ^2^ = 6.445], and multiple lesions (P = 0.036; χ^2^ = 4.404) were significantly associated with poorer OS. Abnormal lymphoma cells in the CSF were an independent predictor of poor prognosis on multivariate analysis (P = 0.034; hazard ratio (HR) = 2.836; 95% confidence interval, 1.082–7.434). Our results indicate that pemetrexed plus lenalidomide is effective for heavily treated R/R PCNSL, with moderate toxicity. Trial registration: #ChiCTR1900028070.

## Introduction

Primary central nervous system lymphoma (PCNSL) is an invasive extranodal lymphoma localized in the brain parenchyma, spinal cord, cerebrospinal fluid (CSF), or vitreoretinal space, with no evidence of systemic lymphoma at initial diagnosis (1). The incidence of PCNSL is 0.4–0.5 per 100,000 individuals annually, accounting for 4% of intracranial malignant tumors and 4%–6% of extranodal lymphomas (2). Regarding pathological type, more than 90% of patients with PCNSL have diffuse large B-cell lymphoma (DLBCL), and the majority originating from non-germinal center B-cells (non-GCBs) (3). Due to the rarity of this disease in the relapsed/refractory setting, there are no large-scale randomized clinical trials, and limited treatment options exist. At present, chemotherapy with high-dose methotrexate (HD-MTX) is generally recommended as the first-line treatment for PCNSL, with an overall response rate (ORR) of 70%–87% and a 2-year overall survival (OS) rate of 58%–67% (4, 5); however, approximately 10%–30% of patients have primarily refractory disease and 50% of patients relapse after remission (6, 7). Although various treatment options, including chemotherapy, radiotherapy, and autologous hematopoietic stem cell transplantation (ASCT), have been evaluated for use in patients with relapsed/refractory (R/R) PCNSL (8–14), a standard treatment strategy has yet to be established and patient prognosis remains extremely poor. These studies treatments revealed heterogeneous survivals (range, 2-59) months.

Pemetrexed, similar to methotrexate, is an antifolate agent that contains a pyrrolidine core that exerts anti-tumor effects by disrupting the normal intracellular floate dependent metabolic process. Lenalidomide is a novel immunomodulator with immunoregulatory and antitumor functions that shows preferential anti-lymphoma activity against non-GCB subtypes of systemic DLBCL (15). Previous studies have reported that pemetrexed (13, 16, 17) or lenalidomide (12, 18, 19) alone, or in combination with other agents, are active drugs in patients with R/R PCNSL. Both drugs can penetrate the blood-brain barrier. Pemetrexed has been reported to be involved in the activation of NF-κB signaling, which may induce pemetrexed resistance, and lenalidomide could block NF-κB signaling. We hypothesize that lenalidomide can overcome pemetrexed resistance and the combination of these two drugs may have a synergistic effect. Therefore, we conducted this prospective phase II study to determine the efficacy and safety of pemetrexed combined with lenalidomide for treating patients with R/R PCNSL.

## Methods

### Patients

Thirty-eight patients admitted to our institution with a confirmed diagnosis of R/R PCNSL from January 2018 to June 2020 were enrolled in this study.

### Inclusion and Exclusion Criteria

#### Inclusion Criteria

The inclusion criteria were: 1) age of 18–75 years; 2) confirmed CNS or vitreoretinal involvement with lymphoma; 3) histologically confirmed DLBCL diagnosis confirmed through histopathology and immunohistochemical staining; 4) no evidence of systemic involvement based on imaging; 5) disease progression or recurrence after having undergone at least two different therapeutic regimens before recruited in the trial, including HD-MTX (3.5–8 g/m^2^) based chemotherapy as the primary regimen, with or without whole-brain radiotherapy (WBRT) and/or ASCT.

#### Exclusion Criteria

Exclusion criteria were: 1) Current or prior systemic evidence of lymphoma; 2) severe cardiac insufficiency (New York Heart Association class III or IV); 3) severe liver insufficiency (alanine or aspartate transferase to levels 2-fold higher than the upper normal limit); 4) renal insufficiency (serum creatinine levels higher than the upper normal limit); 5) allergies or contraindications pemetrexed or lenalidomide; 6) other malignant tumors or serious uncontrolled concurrent disease; 7) active hepatitis A, B, or C, or tuberculosis infection, or other causes of immunosuppression; 8) active pregnancy or lactation; 9) participation in other clinical trials within one month.

#### R/R PCNSL Definition

In this study, relapsed PCNSL was defined as biopsy-proven PCNSL initially with complete response (CR) but presence of new lesions. Refractory PCNSL was defined as the presence of stable disease (SD) or progressive disease (PD) after receiving two or more treatment regimens. With regard to the imaging examinations performed, patients with relapsed PCNSL were evaluated with contrast-enhanced magnetic resonance imaging (MRI) or whole-body positron emission tomography-computed tomography.

### Study Design and Regimen

This study was conducted in accordance with the Declaration of Helsinki and Good Clinical Practice guidelines and approved by the Ethics Committee of our hospital. This was registered study on the Chinese Clinical Trial Registry website (#ChiCTR1900028070). All patients provided written informed consent. Pemetrexed [900 mg/m^2^, intravenous infusion, day (d) 1] and lenalidomide (25 mg/day, orally, d1–21) were administered in a 28 day cycle. Additionally, folic acid (5 mg, orally, three times daily), vitamin B12 (1 mg supplementation, intramuscular injection, on the day before pemetrexed), and dexamethasone (15 mg, intravenous infusion, d1–3) were administered to reduce the toxicity of pemetrexed, and aspirin (100 mg/day, orally, d1–21) was given to prevent lenalidomide-induced thrombosis.

### Primary and Secondary Endpoints and Evaluation of Response Efficacy and Adverse Effects

Our trial treatment continued until lymphoma progression or intolerable toxicity developed. Treatment continued to a maximum of eight cycles. The primary endpoint was ORR (CR + CRu + PR), and patients were required to complete at least one cycle of the study regimen to be evaluable. According to International PCNSL Collaborative Group criteria, ORR includes CR, unconfirmed CR (CRu), and partial response (PR) (20). Secondary endpoints included progression-free survival (PFS) and OS. Patient response to PCNSL treatment was assessed after every cycle. Assessments included contrast-enhanced MRI of the brain parenchyma and spinal cord, lumbar puncture of CSF, and detailed ophthalmic examination of eyes. After each cycle, patient’s condition was observed, and blood tests were performed routinely. In accordance with the National Cancer Institute Standard for Common Terminology Criteria for Adverse Events version 4.0, adverse events (AEs) were assessed as grade 1–5, with each grade indicating mild, moderate, severe, life-threatening, or death, respectively.

### Statistical Analysis

We used the PASS 15 statistical software for single-stage phase II clinical trials to calculate the sample size for this study. Assuming α = 0.05, β = 0.1, power = 0.9, P0 = 0.25 (where P0 is the maximum response proportion to a poor treatment), and P1 = 0.5 (where P1 is the minimum response proportion to a good treatment), the minimum sample size was estimated to be 33. Estimating a dropout rate of 10%, the final enrollment was calculated to 38. Medians, ranges or proportions were used to describe patient clinical baseline characteristics. PFS and OS were calculated using Kaplan-Meier curves. Univariate and multivariate prognostic analyses were performed using the log-rank test and a Cox regression model, respectively. Statistical significance was determined from two-tailed P-values and 95% confidence intervals (CI), where P-values < 0.05 indicated statistically significant differences. SPSS 26.0 (IBM Company, USA) and GraphPad Prism 7 software (Insightful Science Company, San Diego, California, USA) were used for data analyses.

## Results

### Patient Baseline Characteristics

**Figure 1:**
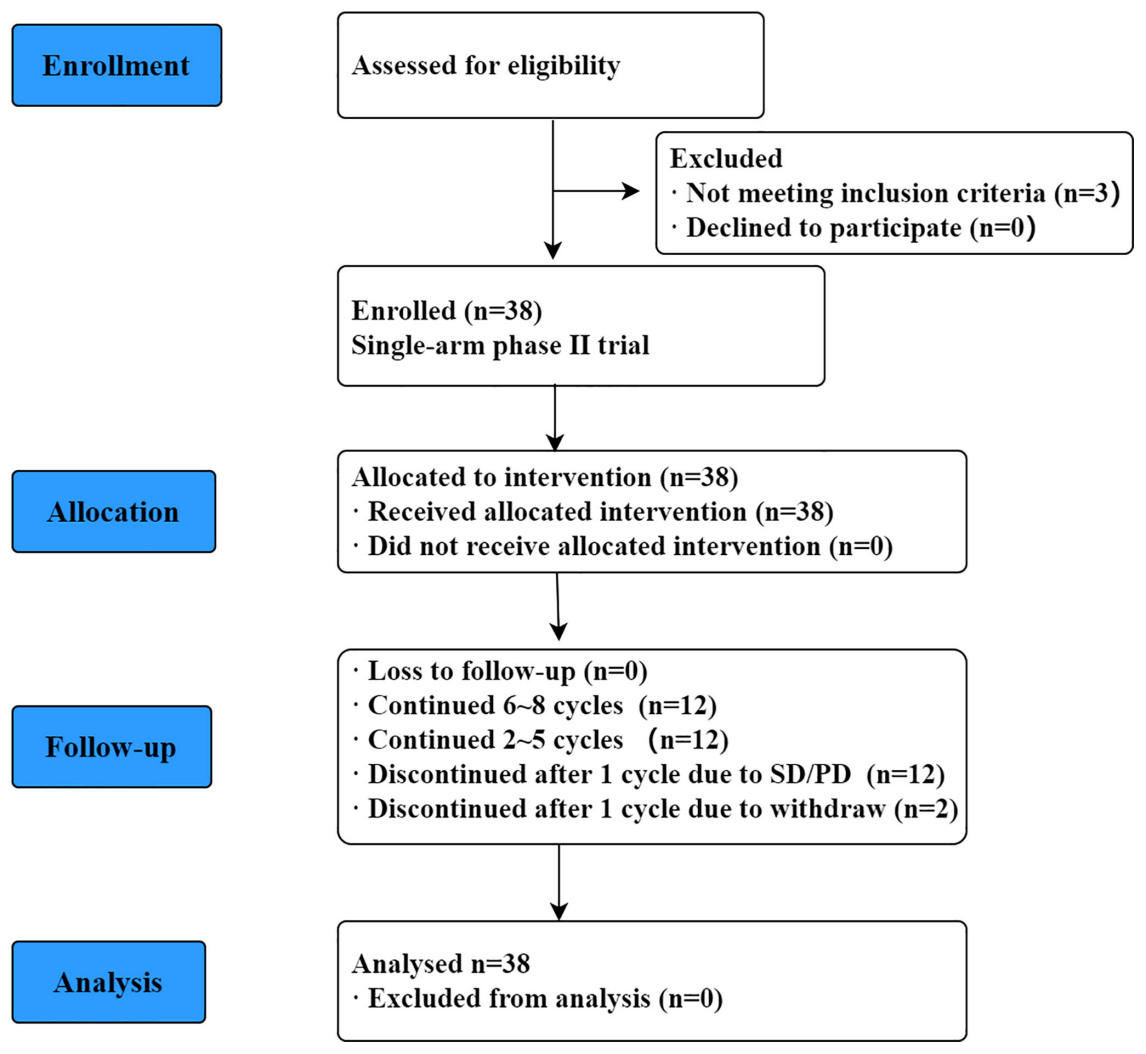
CONSORT flowchart of R/R PCNSL patients in the study.

**Figure 1** is a CONSORT flowchart illustrating the process of patient inclusion in our study. A total of 38 patients with R/R PCNSL were enrolled in our study from January 2018 to June 2020, and their median age was 57 (range, 33–73) years. The clinical baseline characteristics of the patients are shown in **Table 1**. Eastern Cooperative Oncology Group (ECOG) score was 1–2 for 17 patients (44.7%) and 3–4 for 21 patients (55.3%). At the time of inclusion, 21 (55.3%) and 17 (44.7%) patients were diagnosed as having relapsed and refractory PCNSL, respectively. Of all patients, 73.7% experienced recurrence or progression within 12 months of initial diagnosis. Patients underwent a median of 3 (range, 2–10) prior treatment regimens to enrollment. Twenty-four patients (63.2%) had previously undergone WBRT, and three patients received ASCT in addition to WBRT. Eighteen patients had only recurrence/progression involving brain parenchyma, 12 patients had brain parenchymal involvement and leptomeningeal abnormalities, and 5 patients had brain parenchymal and vitreoretinal involvement. A further 3 patients presented with only leptomeningeal abnormalities, and one patient presented with only spinal cord lesions. Other characteristics, such as focality, histology pathological, GCB or non-GCB subtype, deep lesions, and bulky disease were shown in **Table 2**.

**Table 1:** Baseline characteristics of all patients (N = 38).

Clinical characteristics	Patients (N)	Percentage %
Age, years		
Median (range)	57 (33-73)	
Gender		
Male	26	68.4
Female	12	31.6
ECOG score		
1-2	17	44.7
3-4	15	55.3
Type of disease at inclusion		
Relapse	21	55.3
Refractory	17	44.7
Time to relapse/progression		
≤12 months	28	76.3
>12 months	10	23.7
Previous treatment		
Type of Previous regimens≥2	38	100
Median (range)	3 (2-10)	
2	16	42.1
3	11	28.9
4	5	13.2
≥5	6	15.8
Previous MTX based regimens	38	100
Previous Ara-C based regimens	36	94.7
Previous WBRT	24	63.2
Previous ASCT	3	7.9
Previous ASCT+ WBRT	3	7.9
Site of tumor lesion		
Brain parenchymal only	18	47.3
Brain + eyes	5	13.2
Brain + leptomeningeal	12	31.6
Leptomeningeal only	3	7.9
Spinal cord + leptomeningeal	1	2.6
Eyes only	0	0
Focality		
Single focus	17	44.7
Mutifocal	21	54.3
GCB subtype		
GCB	8	21.1
Non-GCB	8	21.1
Unclassified	22	57.8
Histology subtypes		
DLBCLs	38	100
Deep lesion^1^	23	60.5
Bulky disease^2^	17	44.7

ECOG, eastern cooperative oncology group; MTX, methotrexate; Non-GCB, non-germinal center B-cell; WBRT, whole-brain radiology therapy; ASCT, autologous hematopoietic stem-cell transplantation; DLBCLs, diffuse large B-cell lymphomas.

Deep lesion^1^ defined as lesions located more than 3cm from the brain surface; Bulky disease^2^ refers to the lesion diameter greater than 3cm.

**Table 2:** Treatment efficacy observation (N = 38).

Characteristic	ALL (Number = 38)	Percent (%)
ORR	26	68.4
CR	15	39.5
CRu	6	15.8
PR	5	13.1
SD	8	21.1
PD	4	10.5

ORR, overall survival rate; CR, complete response; CRu, unconfirmed complete response; PR, partial response; SD, stable disease; PD, progressive disease.

### Treatment Response and Survival

All 38 patients received the trail regimen, of which 12 patients completed 6-8 cycles 12 patients continued 2-5 cycles to SD/PD, 12 patients discontinued after one cycle due to SD/PD, and 2 patients discontinued after one cycle due to withdrew. Median number of cycles was 3 (range, 1-8).

All patients were evaluated. As shown in **Table 2**, the best responses were observed in 15 patients (39.5%) with CR, while 6 (15.8%) achieved CRu, 5 (13.1%) with PR, 8 (21.1%) with SD, and 4 (10.5%) with PD, resulting in an ORR of 68.4% and disease control rate (CR + PR + SD) of 89.5% (**Table 2**).

**Figure 2:**
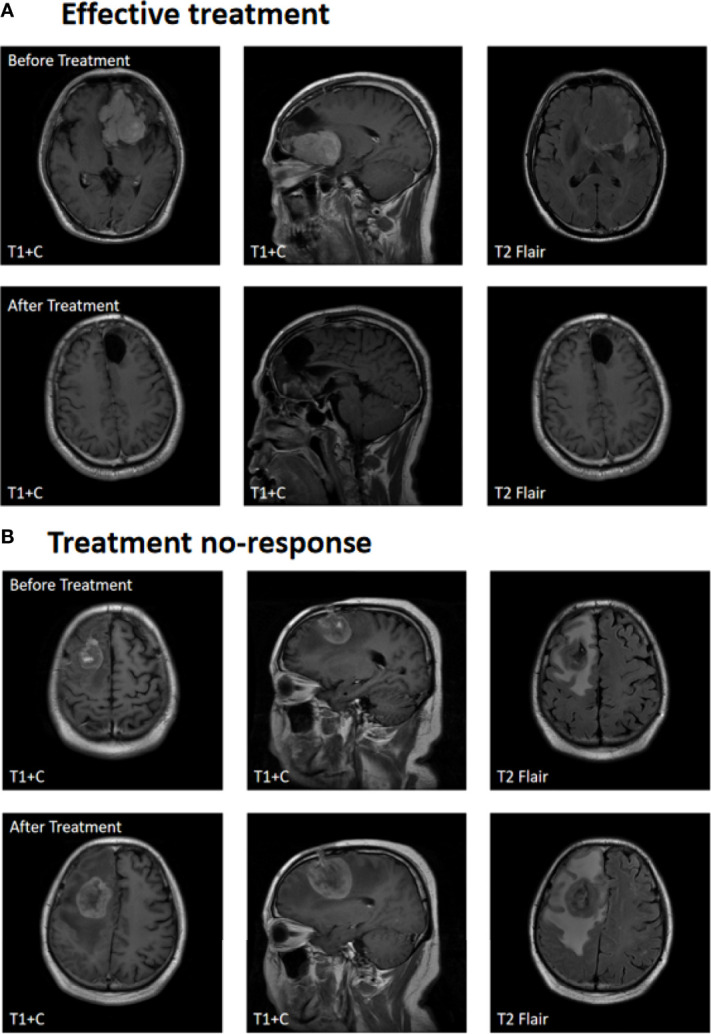
Treatment response. **(A)** Effective treatment. **(B)** Treatment no-response.

**Figure 3:**
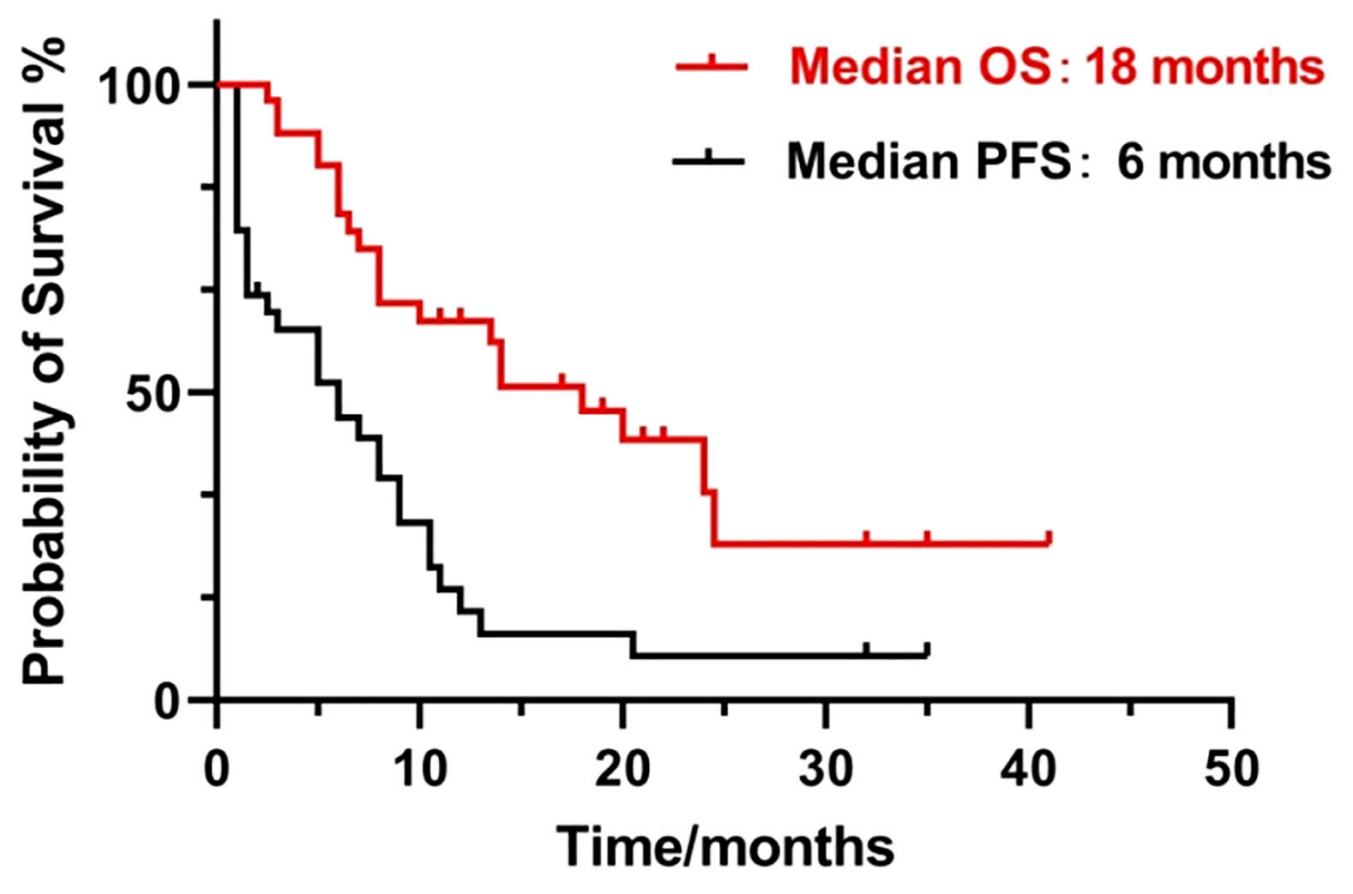
Kaplan-Meier survival curve of PFS and OS time of 38 patients.

**Figure 2A** shows contrast-enhanced MRI findings (before and after treatment) of a sample patient who had an effective treatment response and **Figure 2B** shows the contrast-enhanced MRI findings of a patient with no response. After treatment, the median PFS time was 6 months, with 6-month PFS rates, and the 12-month PFS rates were 50% and 10.5%, respectively. The median OS time was 18 months, and the 1-year and 2-year overall survival rates were 52.6% and 13.2%, respectively (**Figure 3**).

### Safety

Common AEs included fatigue, nausea, fever, infection, and myelosuppression. Five patients developed fatigue (grade 1, 4 cases and grade 2, 1 case). Three patients developed nausea (grade 1, 2 cases and grade 2, 1 case). Four patients developed fever (grade 1, 3 cases and grade 2, 1 case). Four patients developed infection (grade 1, 3 cases and grade 5, 1 case), one of which grade 5 died of pneumonia. Myelosuppression mainly included leukopenia (grade 1, 6 cases and grade 3, 1 case), neutropenia (grade 1, 6 cases and grade 3, 2 cases), anemia (grade 1, 8 cases), and thrombocytopenia (grade 2, 1 case and grade 3, 3 cases). Other AEs included cardiac disorder (grade 1, 2 cases) and thrombogenesis (grade 2, 3 cases). No incidence of vomiting, abnormal liver and kidney functions, constipation, or leukoencephalopathy was noted (**Table 3**).

**Table 3:** Adverse events (N = 38).

Toxicity	All Grades	>Grade3
Fatigue	5	0
Nausea	3	0
Vomiting	0	0
Constipation	0	0
Fever	4	0
Infection	4	1^*^
Cardiac disorder	2	0
ALT/AST increased	0	0
Creatinine increased	0	0
Anemia	8	0
Leukopenia	7	0
Neutropenia	8	0
Thrombocytopenia	5	0
Leukoencephalopathy	0	0
Thrombogenesis	3	0

ALT, alanine aminotransferase; AST, aspartate aminotransferase.

1*grade5 (2.63%).

### Analysis of Prognostic Factors

**Figure 4:**
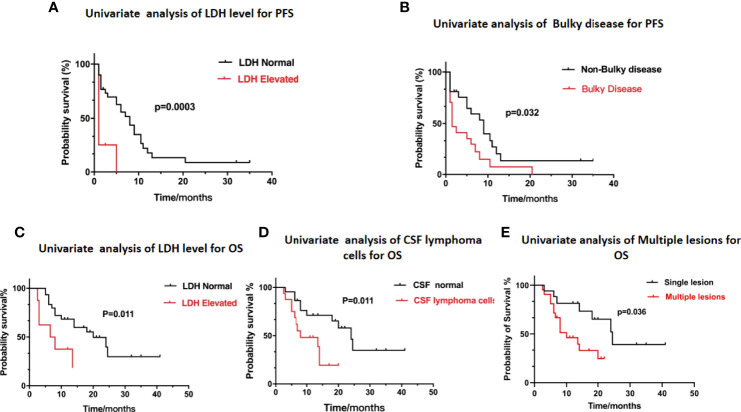
Kaplan-Meier survival curve and prognosis analysis. **(A)** Univariate analysis of LDH level for PFS. **(B)** Univariate analysis of Bulky disease for PFS. **(C)** Univariate analysis of LDH level for OS. **(D)** Univariate analysis of CSF lymphoma cells for OS. **(E)** Univariate analysis of Multiple lesions for OS.

Univariate analysis of the PFS and OS of patients with R/R PCNSL was performed using the log-rank test. As shown in **Table 4**, elevated serum lactate dehydrogenase (LDH) levels (P = 0.0003; χ^2^ = 13.25) and bulky disease (refers to the lesion diameter greater than 3cm) (P = 0.032; χ^2^ = 4.580) were significantly related to shorter PFS (**Figures 4A, B**). Elevated serum LDH levels (P = 0.011; χ^2^ = 6.560), abnormal lymphoma cells in the CSF (P = 0.011; χ^2 =^ 6.445), and multiple lesions (P = 0.036; χ^2^ = 4.404) were significantly associated with poor OS (**Figures 4C–E**). Multivariate Cox regression analysis for OS was performed to select prognostic factors with statistically significant differences as per univariate analysis (P < 0.05), and the results showed that abnormal lymphoma cells in the CSF was an independent poor prognostic factor [P = 0.034; HR = 2.836; 95% CI, 1.082–7.434] (**Table 5**).

**Table 4:** Univariate analysis of PFS and OS time in 38 patients with R/R PCNSL.

Clinical characteristics	MedianPFS	HR 95%CI	χ^2^	P value	MedianOS	HR 95%CI	χ^2^	P value
Age								
<60 years	5	1.098 (0.516~2.339)	0.174	0.676	13.5	1.405 (0.589~3.381)	0.598	0.439
≥60 years	8				24.5			
Gender								
Female	6	0.827 (0.371~1.842)	0.293	0.588	10.75	1.958 (0.753~5.093)	2.523	0.112
Male	5				24			
ECOG								
3-4	6	1.127 (0.527~2.409)	0.260	0.611	14	1.013 (0.427~2.403)	0.001	0.976
1-2	6				18			
LDH								
Elevated level	1	16.00 (3.469~73.78)	13.25	0.0003	7.25	6.560 (1.596~26.96)	6.504	0.011
Normal level	8				20			
GCB subtype								
GCB	7	0.857 (0.297~2.47)		0.661	Undefined	0.186 (0.032~1.095)	3.458	0.063
Non-GCB	6				14			
Eyes involvement								
Yes	3	1.002 (0.326~3.088)	0.568	0.451	24	2.721 (1.058~6.997)	1.750	0.186
No	7				14			
Deep lesion								
Yes	6	1.159 (0.572~2.349)	0.187	0.665	13.5	1.839 (0.781~4.329)	2.482	0.115
No	7				24			
Bulky disease								
Yes	1.5	2.494 (1.114~5.585)	4.580	0.032	14	0.942 (0.393~2.260)	0.082	0.775
No	9				18			
Prior WBRT								
Yes	2.5	1.755 (0.768~4.008)	2.925	0.087	14	1.789 (0.647~4.946)	0.972	0.324
No	8				Undefined			
CSF lymphoma cells								
Yes	5	1.842 (0.860~3.945)	3.778	0.052	8	2.721 (1.058~6.997)	6.445	0.011
No	9				24			
CSF protein level								
Elevated	5	2.112 (0.966~4.617)	3.505	0.061	13.5	2.296 (0.940~5.609)	3.606	0.058
Normal	7				24			
Lesion								
Single lesion	8	0.709 (0.350~1.436)	1.080	0.299	18	0.388 (0.164~0.920)	4.404	0.036
Multiple lesions	5				8			
Type of disease at inclusion								
Refratory	9	1.556 (0.726~3.335)	0.487	0.485	14	1.047 (0.444~2.468)	0.013	0.910
Relapse	5				20			

PFS, progression free survival; OS, overall survival; ECOG, eastern cooperative oncology group; LDH, elevated lactic dehydrogenase; Non-GCB, non-germinal center B-cell; CSF, cerebrospinal fluid; WBRT, whole-brain radiology therapy.

**Table 5:** Multivariate Cox regression analysis of OS for 38 patients with R/R PCNSL.

Clinical Factors	Std. Err.	Z value	*P* value	HR 95%CI
CSF abnormal	0.492	4.493	0.034	2.836 (1.082~7.434)
LDH>250 U/L	0.541	3.594	0.058	2.791 (0.966~8.063)
Multiple lesions	0.847	2.380	0.123	2.334 (0.795~6.849)

HR, hazard ratio; 95% CI, 95% confidence interval.

## Discussion

Although various salvage treatments, including chemotherapy, radiotherapy, and ASCT are available for patients with R/R PCNSL, a standard effective treatment has not been established, and the prognosis remains unsatisfactory. Due to the rarity of the disease, no data from randomized phase III trials on R/R PCNSL have been reported; however, without effective treatment, the OS of patients with R/R PCNSL is ≤ 2 months (21). Previous studies have reported that pemetrexed (alone or in combination with other agents) (13, 16, 17) or lenalidomide (alone or in combination with other agents) (12, 18, 19) are active drugs in patients with R/R PCNSL; however, long-term survival is observed only in a few patients. Pemetrexed has been reported to be involved in the activation of NF-κB signaling, which may induce pemetrexed resistance, and lenalidomide could block NF-κB signaling. Both drugs can penetrate the blood-brain barrier. We hypothesize that lenalidomide can overcome pemetrexed resistance and the combination of these two drugs may have a synergistic effect. Therefore, we conducted this prospective phase II study to determine the efficacy and safety of pemetrexed combined with lenalidomide for treating patients with R/R PCNSL.

The primary analysis included all enrolled eligible patients who had received at least one cycle of our study regimen. According to our results, the ORR was 68.4% and the disease control rate was 89.5%. The best response rates were as follows: CR + CRu, 55.3%; PR, 13.1%; SD, 21.1%; and PD, 10.5% (**Table 2**). Notably, some patients who had relapsed or progressed after received four or more regimens, still demonstrated good response in our study regimen. After treatment, the median PFS and OS were 6 and 18 months, respectively (**Figure 3**). Patients who did not respond or progress would be rechallenged with HD-MTX based regimens (when MTX interval >1year), or other regimens, including BTKi, Pomalidomide, PD-1, or WBRT. These treatments may have prolonged the survival. Our results are comparable or superior to those of other chemotherapeutic agents used for the treatment of R/R PCNSL. Several studies evaluated pemetrexed monotherapy or in combination with other drugs in the treatment of R/R PCNSL, with an OS rate of 55-62.9%, a median PFS time of 4.2-7.8 months, and a median OS time of 7.8-19.5 months (16, 17, 22, 23). Caroline et al. (19) first reported lenalidomide as salvage treatment for relapsed PCNSL, 2 of 6 patients achieved a confirmed CR and one patient achieved PR. A prospective phase II study (18) reported a combination of lenalidomide and rituximab treatment for R/R PCNSL with an ORR of 64.7%, the median PFS and OS were 7.8 months and 17.7 months. Our study regimen shows an advantage response rate over pemetrexed or lenalidomide monotherapy, and was comparable to pemetrexed or lenalidomide in combination with other chemotherapeutic agents used for the treatment of R/R PCNSL.

Prospective studies using other agents, such as topotecan, temozolomide, and rituximab, have shown ORR of 31%–55% and PFS of 1.6–5.7 months (24–26). Previous studies have also reported that intensive chemotherapy followed by ASCT can be effective against R/R PCNSL. Few studies reported the effect of ASCT for R/R PCNSL, with a 2-year OS rate of 45-68%, 2-year-PFS rate of 43-54%, and a median OS of 18.3-86months (27–29). In recent years, several studies with novel targeted drugs that show efficacy in R/R PCNSL have been conducted, including the use of Bruton’s tyrosine kinase inhibitors [ibrutinib (30, 31) and tirabrutinib (10)], mTOR inhibitors [temsirolimus (32)], immunomodulatory drugs [pomalidomide (33)], and immune checkpoint inhibitors [anti-PD1 monoclonal antibodies (34)]. An increasing number of these drugs are being evaluated for use in patients with R/R PCNSL, some of which have been incorporated into the National Comprehensive Cancer Network guidelines.

Moderate toxicity was observed in patients enrolled in this clinical study. The most common AEs included fatigue, nausea, fever, infection, and myelosuppression. Other rare AEs included cardiac dysfunction and thrombosis. One patient died of pneumonia (grade 5). There were no other AEs, such as abnormalities of liver and kidney function, constipation, or leukoencephalopathy (**Table 3**). Treatment with granulocyte colony stimulating factor and thrombopoietin corrected myelosuppression from treatment. Patients with thrombosis, which manifested as deep vein thrombosis, responded to anticoagulant therapy. The low number of AEs was likely related to effective early prevention and dynamic monitoring. We also compared our safety data with other trials using pemetrexed or lenalidomide and found that the two-drug combination did not increase unexpected toxicity.

Currently, there are two widely used prognostic models for PCNSL: Memorial Sloan Kettering Cancer Center (MSKCC) (35) and International Extranodal Lymphoma Study Group (IELSG) (36). The MSKCC prognostic score includes age and Karnofsky performance status and divides patients into three different prognostic classes. The IELSG prognostic score system includes five parameters: age, ECOG score, serum LDH level, protein level in the CSF, and deep brain lesions. We also performed univariate and multivariate analysis for the 38 patients utilizing these and other factors. Univariate analysis showed that elevated serum LDH level and bulky disease were poor prognostic factors for PFS. Elevated serum LDH level, abnormal lymphoma cells in the CSF, and multiple lesions were significantly related to poor OS. Multivariate survival analysis for OS showed that abnormal lymphoma cells in the CSF was an independent prognostic factor [P = 0.034; HR = 2.836; 95% CI, 1.082–7.434].

This study is limited by being a single-center study that included a small sample size. In addition, a non-randomized research design was used. Therefore, multicenter, randomized clinical trials are needed to confirm the results. Nevertheless, the trial regimen was well-tolerated and resulted in good PFS indicating that this drug combination may be another option for patients with R/R PCNSL.

## Data Availability Statement

The original contributions presented in the study are included in the article/supplementary material. Further inquiries can be directed to the corresponding author.

## Ethics Statement

The studies involving human participants were reviewed and approved by Ethics Committee of Huashan Hospital, Fudan University. The patients/participants provided their written informed consent to participate in this study.

## Author Contributions

JM, ZL, TD, YM, and BC prepared and conceived the study. JM, ZL, and TD conducted and analyzed the data. JM, YM, and BC interpreted the results. JM wrote the manuscript. All authors discussed the results and revised the manuscript. All authors contributed to the article and approved the submitted version.

## Funding

This prospective phase II study is the part of a research project (SHDC12020112) that was funded by Shanghai Shenkang Clinical Innovation Project. This study was also funded by the Clinical Research Plan (SHDC2020CR6005-002).

## Conflict of Interest

The authors declare that the research was conducted in the absence of any commercial or financial relationships that could be construed as a potential conflict of interest.

## Publisher’s Note

All claims expressed in this article are solely those of the authors and do not necessarily represent those of their affiliated organizations, or those of the publisher, the editors and the reviewers. Any product that may be evaluated in this article, or claim that may be made by its manufacturer, is not guaranteed or endorsed by the publisher.
